# Interpreting Mini-Mental State Examination Differences in Stable Nonhypoxemic Chronic Obstructive Pulmonary Disease: Letter to the Editor

**DOI:** 10.31662/jmaj.2026-0172

**Published:** 2026-06-05

**Authors:** Yudai Kaneda

**Affiliations:** 1 Clinical Training Center, Jyoban Hospital of Tokiwa Foundation, Fukushima, Japan; 2 Health in Humanitarian Crises, London School of Hygiene & Tropical Medicine, London, UK

**Keywords:** chronic obstructive pulmonary disease (COPD), cognitive impairment, Mini-Mental State Examination (MMSE), educational attainment, confounding bias

I read with great interest the study by Agrawal et al. ^(1)^ examining cognitive function in stable, nonhypoxemic patients with chronic obstructive pulmonary disease (COPD), which provides important insights into early cognitive impairment in this population.

However, I would like to raise a concern regarding the potential impact of confounding factors between groups. In this study, a significant imbalance in educational status was observed (27 illiterate individuals in the COPD group vs. 12 in the control group; conversely, only 1 highly educated individual in the COPD group vs. 17 in the control group; p = 0.000082); yet, no adjustment for this factor appears to have been performed. Given that the Mini-Mental State Examination (MMSE) is strongly influenced by educational attainment, individuals with lower educational levels may score lower despite comparable cognitive function, thereby limiting the validity of applying a uniform cutoff across populations with differing educational backgrounds ^(2)^. In addition, a significant difference in age was also observed between the groups (45.95 ± 4.14 vs. 38.65 ± 6.87 years; p < 0.0001), which may further contribute to the observed differences in MMSE scores ^(3), (4)^.

In this context, the approximately 3-point difference in MMSE scores observed in this study (24.65 vs. 27.56) may indeed reflect cognitive impairment associated with COPD; however, it may also be, at least in part, overestimated due to confounding factors such as differences in educational level and age ^(2), (3), (4)^. Careful interpretation of this finding is warranted, and the lack of adjustment for educational attainment and related confounders should be explicitly acknowledged as an important limitation of the study. In light of this, I would appreciate the authors’ clarification on the extent to which pulmonary dysfunction itself is considered an independent contributor to cognitive impairment in this cohort, as such clarification would help to better delineate the relative contributions of disease-specific and sociodemographic factors to cognitive outcomes.

## Article Information

### Author Contributions

Conception and writing – original draft: Yudai Kaneda

### Conflicts of Interest

None

### AI Use Statement

The author used ChatGPT (OpenAI, San Francisco, California, USA) to assist with English language editing and translation of the manuscript. The tool was used solely for linguistic refinement. The author takes full responsibility for the content of the manuscript.
